# Impact of the *CYP3A5*, *CYP3A4*, *COMT*, *IL-10* and *POR* Genetic Polymorphisms on Tacrolimus Metabolism in Chinese Renal Transplant Recipients

**DOI:** 10.1371/journal.pone.0086206

**Published:** 2014-01-21

**Authors:** Chuan-Jiang Li, Liang Li, Li Lin, Hai-Xia Jiang, Ze-Yan Zhong, Wei-Mo Li, Yan-Jun Zhang, Ping Zheng, Xu-Hui Tan, Lin Zhou

**Affiliations:** 1 Department of Surgery, Nanfang Hospital, Southern Medical University, Guangzhou, PR China; 2 Department of Medical Genetics, School of Basic Medical Sciences, Southern Medical University, Guangzhou, PR China; 3 Department of Clinical Laboratory, Nanfang Hospital, Southern Medical University, Guangzhou, PR China; 4 College of Pharmacy, University of Cincinnati Academic Health Centre, Cincinnati, Ohio, United States of America; 5 Department of Pharmacy, Nanfang Hospital, Southern Medical University, Guangzhou, PR China; 6 Department of Biostatistics, School of Public Health and Tropical Medicine, Southern Medical University, Guangzhou, PR China; Universite de Montreal, Canada

## Abstract

Tacrolimus is a widely used immunosuppressive drug for preventing the rejection of solid organ transplants. The efficacy of tacrolimus shows considerable variability, which might be related to genetic variation among recipients. We conducted a retrospective study of 240 Chinese renal transplant recipients receiving tacrolimus as immunosuppressive drug. The retrospective data of all patients were collected for 40 days after transplantation. Seventeen SNPs of *CYP3A5*, *CYP3A4*, *COMT*, *IL-10* and *POR* were identified by the SNaPshot assay. Tacrolimus blood concentrations were obtained on days 1–3, days 6–8 and days 12–14 after transplantation, as well as during the period of the predefined therapeutic concentration range. Kruskal–Wallis test was used to examine the effect of genetic variation on the tacrolimus concentration/dose ratio (*C*_0_/*D*) at different time points. Chi-square test was used to compare the proportions of patients who achieved the target *C*_0_ range in the different genotypic groups at weeks 1, 2, 3 and 4 after transplantation. After correction for multiple testing, there was a significant association of *C*_0_/*D* with CYP3A5*3, CYP3A4*1G and *CYP3A4* rs4646437 T>C at different time points after transplantation. The proportion of patients in the *IL-10 rs1800871-TT* group who achieved the target *C*_0_ range was greater (*p* = 0.004) compared to the *IL-10 rs1800871-CT* and *IL-10 rs1800871-CC* groups at week 3 after transplantation. CYP3A5*3, CYP3A4 *1G, *CYP3A4* rs4646437 T>C and *IL-10* rs1800871 C>T might be potential polymorphisms affecting the interindividual variability in tacrolimus metabolism among Chinese renal transplant recipients.

## Introduction

Tacrolimus is an effective immunosuppressive drug widely used in solid organ transplantation to prevent rejection [1]. It is characterized by a narrow therapeutic range and large inter- and intraindividual variability in its pharmacokinetics [2]. Therefore, daily drug monitoring and dosage adjustment of tacrolimus are widely used so that the concentrations of the drug can be adjusted to achieve the target trough blood concentration (*C*_0_) range [3]. In current clinical practice, it can take several weeks to reach the target *C*_0_ range and transplant recipients experience significant risk of graft rejection or toxicity during this period; so, it is very important to achieve a stable maintenance dose as quickly as possible [4]. However, dose requirement and the length of time required to reach the target *C*_0_ range show significant interindividual and interethnic variability. Full understanding of this mechanism is highly desirable for the patients to improve the therapeutic efficacy and reduce the side effects.

Tacrolimus is metabolized mainly by biotransformation enzymes cytochrome P450 (CYP) 3A4 and 3A5 [3], [5]. The single nucleotide polymorphism (SNP) *6986A>G* in intron 3 of the *CYP3A5* gene, referred to as CYP3A5*3, results in a splicing defect and the absence of protein activity, unlike the A nucleotide with normal protein activity, referred to as CYP3A5*1. Patients carrying at least one CYP3A5*1 allele are named CYP3A5 expressers and those with CYP3A5*3/*3 genotype are named CYP3A5 nonexpressers [6]. It has been shown that CYP3A5 expressers require a higher maintenance tacrolimus dose and longer time to achieve the target tacrolimus *C*_0_ compared to CYP3A5 nonexpressers among organ transplant recipients [7]–[12]. Moreover, a study revealed that the *CYP3A5* rs28365085 T>C might have functional consequence on CYP3A5 activity [13]. Besides the SNPs of *CYP3A5* gene, the functional variants of *CYP3A4* gene may also influence tacrolimus pharmacokinetics. Wang et al. reported that CYP3A4 *22 (rs35599367, *intron 6 C>T*) markedly affects CYP3A4 mRNA level and could serve as a biomarker for predicting response to CYP3A4-metabolized drugs [14]. The *CYP3A4* rs33972239 delT locates in exon 13 of *CYP3A4* gene. So it is a susceptible variant affecting the enzyme activity. He et al. reported that CYP3A4*1G (rs2242480, *20230 C>T*) allele can increase the activity of the CYP3A4 enzyme [15]. In addition, schirmer et al. reported that *CYP3A4* rs4646437 T>C can affect the hepatic CYP3A4 protein expression levels [16]. Cytochrome P450 oxidoreductase (*POR*) is required for drug metabolism by all microsomal cytochrome P450 enzymes. Zhang et al. reported that SNPs in the *POR* gene influence the rates of P450-mediated drug metabolism in patients [17]. Other studies reported that *POR* rs1057868 C>T and *POR* rs2868177 A>G are associated with CYP3A activity [17], [18]. These SNPs associated with the CYP3A function might influence tacrolimus pharmacokinetics. In a multicenter study, Jacobson et al. reported that rs2239393 A>G and rs4646312 T>C of catechol-*O*-methyltransferase (*COMT*) gene are associated with variation of tacrolimus *C*_0_ */D* [19]. This information suggests that the genetic polymorphisms of *COMT* gene may also affect tacrolimus metabolism. Interleukin-10 (*IL-10*) can regulate CYP3A enzyme activity. It is reported that the administration of IL-10 down-regulated CYP3A activity by 12% in healthy subjects [20]. Thus, the CYP3A-dependent tacrolimus metabolism may be influenced by *IL-10* gene polymorphisms. In addition to the genetic mechanism, clinical factors associated with tacrolimus pharmacokinetics have been reported [21].

Although several factors have been confirmed to impact on tacrolimus pharmacokinetics, some factors with the potential to influence tacrolimus metabolism need to be investigated, especially in different ethnic groups. The aim of this retrospective study was to evaluate the influence of *CYP3A4*, *CYP3A5*, *COMT*, *IL-10* and *POR* SNPs on *C*_0_/*D* and the length of time required to reach the target *C*_0_ range during the early phase after transplantation in a group of Chinese renal transplant recipients.

## Materials and Methods

### Study Design and Patient Population

The study protocol was approved by the Ethical Committee of Nanfang Hospital, an affiliate of the Southern Medical University, China. Written informed consent was obtained from all recipients before their participation in the study. The retrospective study population, from the Nanfang Hospital in Guangzhou, consisted of the renal transplant recipients who received tacrolimus as immunosuppressant between January 2007 and August 2012. Patients with conditions that could affect tacrolimus pharmacokinetics and pharmacodynamics were excluded. Exclusion criteria were hepatitis B (58 patients), hepatitis C (6), cancer (5), systemic lupus erythematosus (SLE) with long-term hormone therapy (4), liver and renal transplantation (7), second renal transplantation (10), acute rejection (5), <18 years old (2). Finally, a total of 240 patients were eligible for the retrospective study. Demographic characteristics, laboratory test results and drug administration history were extracted from electronic medical records. The retrospective data of all patients were collected for 40 days after transplantation.

### Immunosuppressant Regimens and Tacrolimus Measurement

All patients were treated with a combination of immunosuppressants consisting of tacrolimus, mycophenolate mofetil and steroids. The first oral administration of tacrolimus was given approximately 12 h after the transplantation. The initial dosage was calculated according to the weight of the patient (0.10 mg/kg body weight, twice a day) and subsequently adjusted according to the trough blood concentration (*C*_0_), which was measured by the Microparticle Enzyme ImmunoAssay on an IMx analyzer (Abbott Laboratories, Chicago, IL). Patients' *C*_0_ were measured every other day after transplantation during hospitalization and twice a week after they were discharged from the hospital. The predefined *C*_0_ range was 10–12 ng/ml, and the stable maintenance tacrolimus dose was the dosage at which the target *C*_0_ range was achieved for more than 2 consecutive days and following *C*_0_ values were within the range 9–14 ng/ml. This dosage did not change and was considered to be the stable maintenance tacrolimus dose. The length of time required to reach the target *C*_0_ range was the period from transplantation to the time when patients achieved the stable maintenance tacrolimus dose *D*. *C*_0_ concentration was dose-corrected (*C*_0_/*D*) using the corresponding 24 h dose on a mg/kg basis. *C*_0_/*D* on days 1–3, 6–8 and 12–14 after transplantation, as well as the period of the predefined tacrolimus therapeutic range were selected as the representative ratio parameters of the early phase after transplantation. The corresponding laboratory parameters including hemoglobin, hematocrit, albumin, alanine aminotransferase, aspartate aminotransferase, total bilirubin and unconjugated bilirubin were obtained. The relationships between representative ratio parameters and the genetic variants were analyzed in this study.

### SNP Genotyping and Linkage Disequilibrium Measurement

Human DNA was extracted from leukocytes in peripheral blood using the TIANamp Genomic DNA Kit (Tiangen Biotech, Beijing, China). The SNPs of the *CYP3A5*, *CYP3A4*, *COMT*, *IL-10* and *POR* genes meeting the following two criteria were selected for our study. (1) It has been reported that the SNPs might affect the corresponding gene activity, or the SNPs are located in the coding region of the gene. (2) The minor allele frequency (MAF) is >5% in the CHB population (data from HapMap). Finally, the *CYP3A5* rs776746 A>G (CYP3A5*3 allele), *CYP3A5* rs28365085T>C, *CYP3A4* rs2242480 C>T (CYP3A4*1G allele), *CYP3A4* rs35599367 C>T (CYP3A4*22 allele), *CYP3A4* rs4646437 T>C, *CYP3A4* rs33972239 delT, *POR* rs1057868 C>T, *POR* rs2868177 A>G, *COMT* rs4646312 T>C, *COMT* rs2239393 A>G, *COMT* rs737865 T>C, *COMT* rs6267 G>T, *COMT* rs4680 G>A, *COMT* rs165599 G>A, *IL-10* rs1800871 C>T, *IL-10* rs1800872 C>A and *IL-10* rs1800896 A>G were analyzed in this study. The genotypes of the 17 SNPs were determined by the SNaPshot assay using the Applied Biosystems Multiplex Kit (Life Technologies Corporation, Shanghai, China) [22].All SNPs of 240 patients tested in this study were successfully genotyped and passed quality control. Haplotypes were inferred by a Bayesian statistical method with the PHASE 2.1 software (Stephens and Donnelly 2003). Reconstructed haplotypes were inserted into the Haploview v. 4.2 program to find *r* ^2^.

### Statistical Analysis

The dose-adjusted tacrolimus trough concentration (*C*_0_/*D*) is the ratio of the measured tacrolimus trough concentration *C*_0_ divided by the corresponding daily tacrolimus dose *D* expressed as mg/kg body weight. All values are expressed as mean ± SD. Sample size and statistical power were evaluated using one-way analysis of variance model (unequal n's) based on nQuery advisor version 7.0 (Statistical Solutions, Cork, Ireland). To account for multiple testing, the Bonferroni correction was applied. *P* values for SNPs less than 0.05/N (N = number of SNPs to be analyzed) were considered as significant. All SNPs identified were tested for deviations from Hardy–Weinberg disequilibrium with the use of a χ^2^ test. The following analyses were used to evaluate the impact of each SNP on *C*_0_/*D* and the length of time required to reach the target *C*_0_ range. *C*_0_/*D* among the three genotypes of these SNPs was compared using the Kruskal–Wallis test. *C*_0_/*D* between the two genotypes of these SNPs was compared using the Mann–Whitney test. SNPs that were associated significantly with *C*_0_/*D* were examined for association with the length of time required to reach the target *C*_0_ range. The proportion of patients who achieved the target *C*_0_ range among the different genotypic groups at different time points was analyzed with the χ^2^ test. All statistical analyses were performed using the SPSS software package (version 13.0, SPSS Inc., Chicago, IL).

## Results

### Patient characteristics and genotype frequencies

A total of 240 renal transplant recipients were included in this retrospective study. Of these, 183 finally achieved the target *C*_0_ range through drug monitoring and dosage adjustment. The other 57 patients who hardly achieved the target *C*_0_ range would undergo further therapy. Of the 17 SNPs, 14 (except *CYP3A5* rs28365085 T>C, CYP3A4*22 and *CYP3A4* rs33972239 delT) were identified in the renal transplant recipients. Finally, the 14 SNPs were analyzed in this study. The allele frequencies of the 14 SNPs in 240 patients were in accordance with Hardy–Weinberg equilibrium, and the same results were found in 183 patients with the stable condition. The demographics, clinical characteristics and genotype frequencies of the patients on days 1–3, 6–8 and 12–14 after transplantation, as well as during the period of the predefined tacrolimus therapeutic range are given in Tables 1 and 2.

**Table 1:** Demographics, clinical characteristics of the Chinese renal transplant recipients.

	Days 1 to 3	Days 6 to 8	Days 12 to 14	Stable condition
	n = 240	n = 240	n = 240	n = 183
Age (years), (mean ± SD)	41.03±12.22	41.03±12.22	41.03±12.22	41.77±12.14
Gender (male/female)	161/79	161/79	161/79	124/59
Body weight (kg) (mean ± SD)	57.94±10.12	57.94±10.12	57.94±10.12	58.25±10.18
Hematocrit (%)	0.353±0.0559	0.339±0.0577	0.303±0.0496	0.309±0.0413
Hemoglobin (g/L)	115.2±19.8	112.8±18.8	100.8±16.4	101.2±14.0
Albumin (g/L)	35.3±4.5	35.9±4.9	37.0±5.3	38.6±4.5
Alanine aminotransferase (ALT), (U/L)	18.0±14.0	27.9±35.6	40.6±45.4	29.7±31.2
Aspartate aminotransferase(AST), (U/L)	19.3±11.6	22.2±16.4	22.5±14.0	17.7±9.0
Total bilirubin (TBIL), (μmol/L)	10.22±4.43	11.19±4.57	9.21±3.70	8.99±3.31
Unconjugated bilirubin(IBIL), (μmol/L)	7.12±3.15	7.80±3.34	6.24±2.69	6.08±2.55
Tacrolimus dose (mg/day)	6.64±1.56	7.02±2.16	7.88±2.97	8.30±3.15
Tacrolimus concentration (ng/ml)	10.8±5.4	10.3±4.0	10.1±3.5	11.0±1.3
Concentration/Dose Ratio (ng/ml)/(mg/kg)	95.5±49.1	95.7±57.0	86.3±52.7	90.8±47.1

**Table 2:** Frequencies of allelic variants in the Chinese renal transplant recipients.

Genotypes (n,%)	Days 1 to14	Stable condition
	N = 240	N = 183
*CYP3A5* (*1/*1, *1/*3, *3/*3)	21/103/116	17/81/85
	(8.75%,42.92%,48.33%)	(9.29%,44.26%,46.45%)
*CYP3A5* rs28365085 T>C (T/T, T/C, C/C)	240/0/0	183/0/0
	(100%,0%,0%)	(100%,0%,0%)
*CYP3A4* (*1/*1, *1/*1G, *1G/*1G)	131/90/19	98/73/12
	(54.58%,37.50%,7.92%)	(53.55%, 39.89%, 6.56%)
*CYP3A4* rs4646437 T>C (T/T, T/C, C/C)	10/80/150	6/63/114
	(4.17%,33.33%,62.50%)	(3.28%,34.42%,62.30%)
*CYP3A4* (*1/*1,*1/*22, *22/*22)	240/0/0	183/0/0
	(100%,0%,0%)	(100%,0%,0%)
*CYP3A4* rs33972239 delT (−/−, −/T, T/T)	240/0/0	183/0/0
	(100%,0%,0%)	(100%,0%,0%)
*POR* rs1057868 C>T (C/C, C/T, T/T)	101/107/32	67/90/26
	(42.08%,44.58%,13.34%)	(36.61%,49.18%,14.21%)
*POR* rs2868177 A>G (A/A, A/G, G/G)	84/104/52	65/85/33
	(35.00%, 43.33%,21.67%)	(35.52%,46.45%,18.03%)
*COMT* rs4646312 T>C (T/T, T/C, C/C)	115/98/27	92/73/18
	(47.92%, 40.83%, 11.25%)	(50.27%,39.89%,9.84%)
*COMT* rs2239393 A>G (A/A, A/G, G/G)	116/96/28	93/71/19
	(48.33%, 40.00%, 11.67%)	(50.82%,38.80%,10.38%)
*COMT* rs737865 T>C (T/T, T/C, C/C)	126/92/22	99/70/14
	(52.50%,38.33%, 9.17%)	(54.10%,38.25%,7.65%)
*COMT* rs6267 G>T (G/G, G/T, T/T)	213/26/1	163/19/1
	(88.75%,10.83%, 0.42%)	(89.07%,10.38%,0.55%)
*COMT* rs4680 G>A (G/G, G/A, A/A)	133/86/21	97/69/17
	(55.42%, 35.83%, 8.75%)	(53.01%,37.70%,9.29%)
*COMT* rs165599 G>A (G/G, G/A, A/A)	54/138/48	38/106/39
	(22.50%, 57.50%, 20.00%)	(20.77%,57.92%,21.31%)
*IL-10* rs1800871 C>T (C/C, C/T, T/T)	15/111/114	8/84/91
	(6.25%, 46.25%, 47.50%)	(4.37%,45.90%,49.73%)
*IL-10* rs1800896 A>G (A/A, A/G, G/G)	217/23/0	168/15/0
	(90.42%, 9.58%, 0%)	(91.80%,8.20%,0%)
*IL-10* rs1800872 C>A (C/C, C/A, A/A)	15/112/113	8/85/90
	(6.25%, 46.67%, 47.08%)	(4.37%,46.45%,49.18%)

### Single genetic polymorphism analysis for association with tacrolimus *C_0_/D*

**Figure 1:**
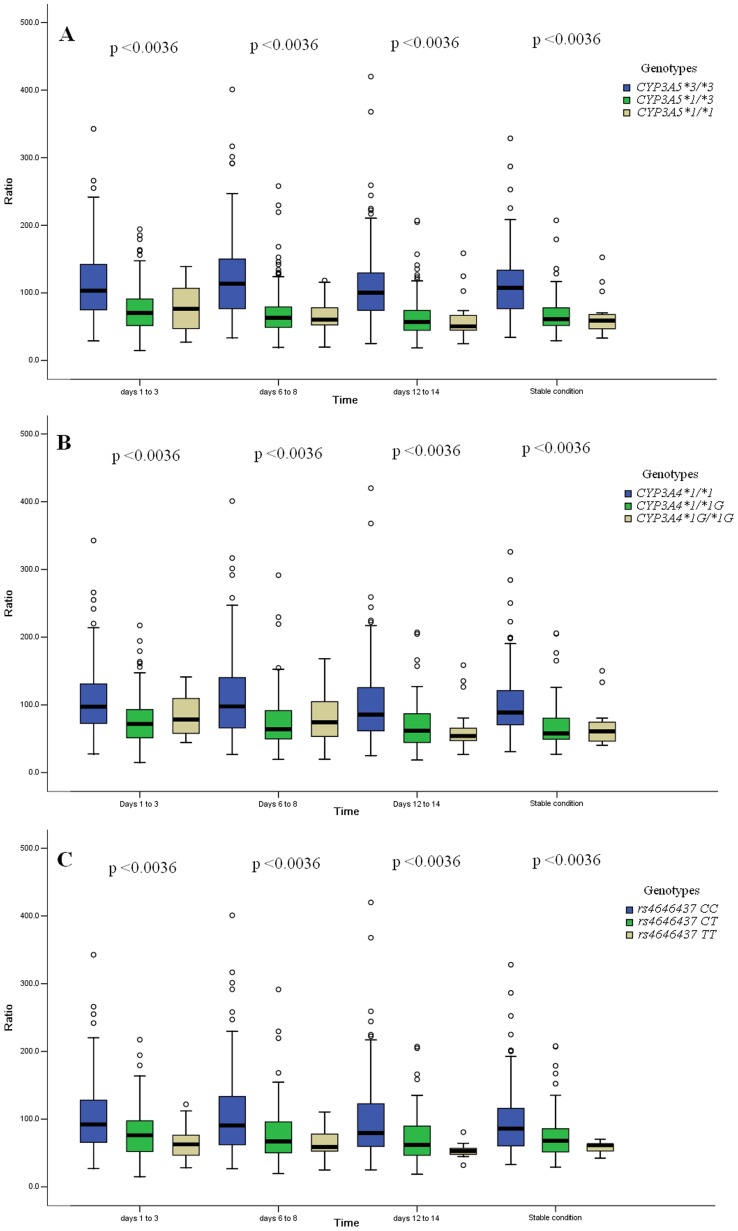
Box-and-whisker plot of tacrolimus *C*_0_/*D* for different genotypic groups. The boxes represent the median, 25th and 75th percentiles of the data. The circles represent deviant cases. The *X*-axis gives the times (days 1–3, 6–8 and 12–14, and the period of stable conditions after transplantation). The *Y*-axis gives the *C*_0_/*D*. Genetic variants are: A, CYP3A5*3; B, CYP3A4*1G; and C, *CYP3A4* rs4646437 T>C. The *p* values among the genetic groups are given above the box-and-whisker plot.

We examined the association of the 14 genotypic variants with tacrolimus *C*_0_/*D* at different time points after transplantation. The level of significance has been adjusted according to the Bonferroni correction (*p*_bonf_ <0.0036). Of the 14 variants, CYP3A5*3, CYP3A4*1G and *CYP3A4* rs4646437 T>C presented a significant association with tacrolimus *C*_0_/*D* at different time points after transplantation (Tables 3 and 4; Figure 1). Tacrolimus *C*_0_/*D* of the patients with CYP3A5 *3/*3 was highest among the different genotypic groups of CYP3A5*3 (Figure 1A). *C*_0_/*D* of the patients with CYP3A4 *1/*1was highest among the different genotypic groups of CYP3A4*1G (Figure 1B). *C*_0_/*D* of the patients with *CYP3A4 rs4646437-CC* was highest among the different genotypic groups of *CYP3A4* rs4646437 T>C (Figure 1C). Moreover, the *IL-10* rs1800871 C>T and *IL-10* rs1800872 C>A presented a marginal association (*p*<0.05) with *C*_0_/*D* at the time point when the patients achieved the maintenance dose (Table 4). However, impact of *IL-10* rs1800871 C>T and *IL-10* rs1800872 C>A on *C*_0_/*D* was not statistically significant after applying Bonferroni correction. None of the other 9 variants demonstrated a significant association with *C*_0_/*D* at any time point. In addition, the minimum sample sizes needed for 80% power for analysis of CYP3A5*3, CYP3A4*1G and *CYP3A4* rs4646437 T>C were estimated, and the sample size (240 patients) is enough to assure the statistical power and conclusion (Table S1).

**Table 3:** Comparison of the tacrolimus *C*_0_/*D* in the different groups classified by genetic variant genotypes.

Genotype	n = 240	(days1 to 3)C_0_/D	p	(days6 to 8)C_0_/D	p	(days 12 to 14) C_0_/D	p
CYP3A5*1/*1	21	78.4±35.1		68.5±27.1		60.7±32.7	
CYP3A5*1/*3	103	76.9±37.1	2.93×10^−9^	72.9±41.2	3.87×10^−13^	65.2±33.7	2.79×10^−14^
CYP3A5*3/*3	116	115.0±53.2		120.9±62.4		109.6±59.3	
CYP3A4*1/*1	131	107.7±53.2		111.2±62.2		100.5±59.7	
CYP3A4*1/*1G	90	79.8±40.4	7.07×10^−5^	76.6±45.0	9.58×10^−7^	69.9±36.6	1.64×10^−6^
CYP3A4*1G/*1G	19	85.6±32.1		79.5±37.3		65.3±35.8	
*CYP3A4* rs4646437 TT	10	68.7±29.2		66.8±25.6		53.8±12.8	
*CYP3A4* rs4646437 TC	80	81.9±40.0	4.32×10^−4^	80.8±47.6	7.41×10^−4^	71.5±39.1	3.48×10^−5^
*CYP3A4* rs4646437 CC	150	104.5±52.3		105.6±60.9		96.3±57.8	
*POR* rs1057868 CC	101	96.2±45.1		95.4±53.6		84.7±52.9	
*POR* rs1057868 CT	107	93.9±52.7	0.714	99.0±61.4	0.422	90.1±54.7	0.444
*POR* rs1057868 TT	32	98.3±50.2		85.8±52.7		78.3±44.9	
*POR* rs2868177 AA	84	94.0±55.6		94.6±62.8		83.9±54.9	
*POR* rs2868177 AG	104	93.8±43.8	0.471	93.2±54.1	0.411	83.4±40.7	0.471
*POR* rs2868177 GG	52	101.1±48.7		102.6±53.6		95.7±68.2	
*COMT* rs4646312 TT	115	95.0±48.5		97.5±55.0		87.5±55.3	
*COMT* rs4646312 TC	98	97.3±53.0	0.994	93.562.9	0.469	84.1±52.5	0.620
*COMT* rs4646312 CC	27	90.9±36.6		96.1±43.1		88.5±42.3	
*COMT* rs2239393 AA	116	95.7±48.6		97.6±54.9		87.8±55.1	
*COMT* rs2239393 AG	96	96.7±53.2	0.966	93.4±63.4	0.413	83.9±53.0	0.577
*COMT* rs2239393 GG	28	90.5±36.0		96.0±42.3		88.0±41.6	
*COMT* rs737865 TT	126	94.7±47.3		95.4±56.2		86.0±57.0	
*COMT* rs737865 TC	92	95.4±53.1	0.749	93.1±60.8	0.153	85.4±48.1	0.632
*COMT* rs737865 CC	22	100.0±43.1		108.1±45.2		91.5±47.3	
*COMT* rs6267 GG	213	94.7±49.1		96.1±59.1		85.7±49.4	
*COMT* rs6267 GT	26	102.2±50.7	0.775	93.8±37.3	0.646	91.2±76.5	0.972
*COMT* rs6267 TT	1	84.5		60.7		74.4	
*COMT* rs4680 GG	133	98.9±52.6		96.4±58.7		87.2±56.5	
*COMT* rs4680 GA	86	90.2±42.7	0.594	94.8±54.0	0.936	84.2±47.1	0.962
*COMT* rs4680 AA	21	95.9±50.8		95.3±61.0		88.4±51.7	
*COMT* rs165599 GG	54	105.0±50.8		103.2±58.0		87.7±43.0	
*COMT* rs165599 GA	138	90.3±45.4	0.117	91.4±58.7	0.144	82.6±56.7	0.089
*COMT* rs165599 AA	48	99.7±55.9		99.6±50.8		95.3±50.4	
*IL-10* rs1800871 CC	15	88.1±40.1		85.4±54.7		75.7±40.2	
*IL-10* rs1800871 CT	111	92.1±47.8	0.330	91.1±58.5	0.104	82.6±57.2	0.129
*IL-10* rs1800871 TT	114	99.7±51.4		101.5±55.8		91.2±49.4	
*IL-10* rs1800896 AA	217	96.3±48.9	0.222	96.7±54.7	0.114	86.6±50.6	0.290
*IL-10* rs1800896 AG	23	88.1±51.5		86.7±76.6		83.2±70.6	
*IL-10* rs1800872 CC	15	88.0±40.1		82.6±55.5		74.0±40.9	
*IL-10* rs1800872 CA	112	92.1±47.8	0.352	91.6±58.0	0.086	83.4±56.8	0.174
*IL-10* rs1800872 AA	113	99.8±51.4		101.6±56.1		90.8±49.7	

**Table 4:** Comparison of the tacrolimus *C*_0_/*D* in the different genotypic groups on the time achieving target blood tacrolimus concentrations.

Genotype	*n* = 183	Stable conditions (*C*_0_/*D*)	*p*
CYP3A5*1/*1	17	67.3±29.9	
CYP3A5*1/*3	81	69.5±27.9	3.01×10^−13^
CYP3A5*3/*3	85	115.7±52.0	
CYP3A4*1/*1	98	105.0±51.4	
CYP3A4*1/*1G	73	74.3±35.5	1.12×10^−6^
CYP3A4*1G/*1G	12	74.6±34.4	
*CYP3A4* rs4646437 TT	6	60.6±9.4	
*CYP3A4* rs4646437 TC	63	78.8±38.8	1.11×10^−3^
*CYP3A4* rs4646437 CC	114	99.0±50.4	
*POR* rs1057868 CC	67	92.4±46.6	
*POR* rs1057868 CT	90	90.9±49.5	0.728
*POR* rs1057868 TT	26	86.1±40.5	
*POR* rs2868177 AA	65	92.0±50.8	
*POR* rs2868177 AG	85	86.5±39.4	0.563
*POR* rs2868177 GG	33	99.4±56.9	
*COMT* rs4646312 TT	92	91.1±48.6	
*COMT* rs4646312 TC	73	87.3±46.0	0.152
*COMT* rs4646312 CC	18	103.2±43.2	
*COMT* rs2239393 AA	93	91.4±48.4	
*COMT* rs2239393 AG	71	87.2±46.5	0.2
*COMT* rs2239393 GG	19	101.1±43.0	
*COMT* rs737865 TT	99	89.6±49.8	
*COMT* rs737865 TC	70	90.2±44.1	0.328
*COMT* rs737865 CC	14	101.4±43.0	
*COMT* rs6267 GG	163	92.3±48.6	
*COMT* rs6267 GT	19	78.1±31.0	0.561
*COMT* rs6267 TT	1	74.4	
*COMT* rs4680 GG	97	89.0±45.5	
*COMT* rs4680 GA	69	94.3±49.9	0.749
*COMT* rs4680 AA	17	86.6±45.9	
*COMT* rs165599 GG	38	94.3±42.1	
*COMT* rs165599 GA	106	89.351.3	0.363
*COMT* rs165599 AA	39	91.3±40.1	
*IL-10* rs1800871 CC	8	73.5±36.8	
*IL-10* rs1800871 CT	84	84.1±46.9	0.017
*IL-10* rs1800871 TT	91	98.4±47.1	
*IL-10* rs1800896 AA	168	90.4±44.5	0.710
*IL-10* rs1800896 AG	15	95.4±72.0	
*IL-10* rs1800872 CC	8	76.5±36.8	
*IL-10* rs1800872 CA	85	84.5±46.6	0.046
*IL-10* rs1800872 AA	90	98.0±47.6	

### Difference in the length of time required to reach the target *C_0_* range

According to the above data, CYP3A5*3, CYP3A4*1G, *CYP3A4* rs4646437 T>C, *IL-10* rs1800871 C>T and *IL-10* rs1800872 C>A might be associated with *C*_0_/*D*. We also evaluated the relationships between the five variants and the length of time required to reach the target *C*_0_ range. The proportion of patients who achieved the target *C*_0_ range was compared for the different genotypic groups at weeks 1, 2, 3 and 4 after transplantation (Table 5). The level of significance has been adjusted according to the Bonferroni correction (*p*_bonf_ <0.01). The proportion of patients in CYP3A4*1/*1 group who achieved the target *C*_0_ range at week 1 was higher (*p* = 0.041) compared to the CYP3A4*1/*1G and CYP3A4*1G/*1G groups. However, the significance was lost after Bonferroni correction. The proportion of patients in the *IL-10 rs1800871-TT* group who achieved the target *C*_0_ range at week 3 was higher (*p* = 0.004) compared to the *IL-10 rs1800871-CT* and *IL-10 rs1800871-CC* groups. There was no significant difference among the other variant groups at any time point.

**Table 5:** The impact of the genetic variants on the time to achieve the target blood tacrolimus concentrations.

	Week 1		Week 2		Week 3		Week 4	
	Stable conditions		Stable conditions		Stable conditions		Stable conditions	
	Yes/No (*n*)	*p*	Yes/No (*n*)	*p*	Yes/No (*n*)	*p*	Yes/No (*n*)	p
CYP3A5*3/*3	9/107	0.058	39/77	0.298	72/44	0.802	79/37	0.369
CYP3A5*1/*3 or *1/*1	3/121		34/90		75/49		91/33	
CYP3A4*1/*1	10/121	0.041	43/88	0.296	79/52	0.855	89/42	0.280
CYP3A4*1/*1G or 1G/*1G	2/107		29/80		67/42		81/28	
*CYP3A4* rs4646437 CC	9/141	0.360	49/101	0.329	95/55	0.307	106/44	0.942
*CYP3A4* rs4646437 TC or TT	3/87		24/66		51/39		64/26	
*IL-10* rs1800871 TT	5/109	0.679	38/76	0.351	77/37	0.004	87/27	0.076
*IL-10* rs1800871 CT or CC	7/119		35/91		62/64		83/43	
*IL-10* rs1800872 AA	5/108	0.700	37/76	0.461	75/38	0.098	86/27	0.091
*IL-10* rs1800872 CA or CC	7/120		36/91		71/56		84/43	

### Linkage between *CYP3A4* SNPs and CYP3A5*3 in tacrolimus metabolism

The *CYP3A4* and *CYP3A5* genes are located in 7q21.1. We analyzed the linkage disequilibrium (LD) between the *CYP3A4* and *CYP3A5* variants. There was a moderate degree of LD between CYP3A4*1/*1G (rs2242480 C>T) and CYP3A5*1/*3 (rs776746 A>G) (*r* ^2^ = 0.502) and a low degree of LD between *CYP3A4* rs4646437 T>C and CYP3A5*1/*3 (rs776746 A>G) (*r* ^2^ = 0.244). We investigated the effect of the CYP3A4*1/*1G and *CYP3A4* rs4646437 T>C polymorphisms on the dose-adjusted tacrolimus concentration (*C*_0_/*D*) among CYP3A5 expressers and nonexpressers (Tables 6 and 7). There was no significant difference in *C*_0_/*D* between patients with the CYP3A4*1G allele and the *1/*1 genotype. The same results were found between patients with the *CYP3A4 rs4646437 T* allele and the *CYP3A4 rs4646437 CC* genotype.

**Table 6:** Tacrolimus *C*_0_/*D* in CYP3A4*1/*1G genotypes classified by different CYP3A5 expressers.

	CYP3A5 expresser		*p*	CYP3A5 nonexpresser		*p*
	CYP3A4*1/*1	CYP3A4*1/*1G+ *1G/*1G		CYP3A4*1/*1	CYP3A4*1/*1G+ *1G/*1G	
N	25	99		106	10	
(days1 to 3) C_0_/D	75.4±37.4	77.6±36.6	0.681	115.3±53.7	112.4±50.0	0.875
(days6 to 8) C_0_/D	73.1±46.0	71.9±37.4	0.988	121.0±62.3	127.9±66.6	0.791
(days 12 to 14) C_0_/D	58.3±23.8	66.0±35.4	0.480	110.5±61.2	100.2±32.1	0.890

**Table 7:** Tacrolimus *C*_0_/*D* in *CYP3A4* rs4646437 genotypes classified by different CYP3A5 expressers.

	CYP3A5 expresser		*p*	CYP3A5 nonexpresser		*p*
	*CYP3A4 rs4646437 CC*	*CYP3A4 rs4646437 TC* + *TT*		*CYP3A4 rs4646437 CC*	*CYP3A4 rs4646437 TC* + *TT*	
N	46	78		104	12	
(days1 to 3) C_0_/D	79.0±37.1	76.1±36.5	0.658	115.8±54.2	108.4±44.9	0.744
(days6 to 8) C_0_/D	70.9±38.7	73.0±39.5	0.668	121.7±62.7	120.4±62.1	0.878
(days 12 to 14) C_0_/D	64.1±28.8	64.6±36.1	0.649	110.6±61.7	101.0±32.0	0.935

## Discussion

This retrospective study examined the contribution of gene polymorphisms to the dose-adjusted tacrolimus concentration (*C*_0_/*D*) and the length of time required to reach the target trough blood concentration range (*C*_0_) in Chinese renal transplant recipients. In accord with the results of earlier studies [7]–[11], we found that CYP3A5*3 presented a significant association (*p*<0.0036) with tacrolimus *C*_0_/*D* at different time points after transplantation (Figure 1A). This result further validated that the CYP3A5*3 allele was strongly associated with tacrolimus pharmacokinetics. In addition, the CYP3A4 *1G allele and *CYP3A4* rs4646437 T>C were associated (*p*<0.0036) with *C*_0_/*D* at different time points after transplantation (Figure 1B and 1C). This is the first report of association between *CYP3A4* rs4646437 T>C and tacrolimus pharmacokinetics. Because the *CYP3A4* and *CYP3A5* genes are both located in 7q21.1, the LD between *CYP3A4* SNPs and *CYP3A5* 6986A>G might influence the impact of *CYP3A4* SNPs on the tacrolimus *C*_0_/*D*. Crettol et al. reported that the *CYP3A4* rs4646437C>T influenced cyclosporine pharmacokinetics, the *rs4646437-T* carriers requiring higher cyclosporine dose. They found also that the *rs4646437-T* allele was in strong LD (*r* ^2^ = 0.82) with the CYP3A5*1 allele in Caucasian renal transplant recipients [23]. In this study, there was a moderate degree of LD between CYP3A4*1/*1G (rs2242480 C>T) and CYP3A5*1/*3 (rs776746 A>G) (*r* ^2^ = 0.502) and a low degree of LD between *CYP3A4* rs4646437 T>C and CYP3A5*1/*3 (rs776746 A>G) (*r* ^2^ = 0.244). Miura et al. reported that the CYP3A4*1/*1G might affect interindividual variability in tacrolimus pharmacokinetics among CYP3A5 expressers [24]. Zuo et al. reported that CYP3A4*1G can influence the oral clearance (CL/F) of tacrolimus in CYP3A5 expressers or nonexpressers among Chinese renal transplant recipients [25]. We divided the patients into CYP3A5 expressers and nonexpressers, and examined the impact of *CYP3A4* variants on *C*_0_/*D* in different CYP3A5 expresser groups. There was no significant difference of *C*_0_/*D* between patients with the CYP3A4*1G allele and the *1/*1 genotype among the different CYP3A5 expresser groups (Table 6). The same result was found between patients with the *CYP3A4 rs4646437-T* allele and the *CYP3A4 rs4646437-CC* genotype (Table 7). This results indicated that the LD with CYP3A5*1/*3 might be one reason for the association between the *CYP3A4* SNPs and *C*_0_/*D* although the LD was not strong in our study population. So, the impact of the two SNPs on tacrolimus metabolism needs further investigation. Zhang et al. reported that liver transplantation recipients with donors who had the *IL-10 rs1800896-AA* genotype had higher *C*_0_/*D* values compared to donors with the *IL-10 rs1800896-AG* genotype [20]. They found also that the *C*_0_/*D* values of liver transplantation recipients with donors who had a low IL-10 production genotype (*rs1800871-TT*, *rs1800872-AA*) were higher compared to a high IL-10 production genotype (*rs1800871-CC* or *CT*, *rs1800872-CC* or *AC*) and they suggested that the expression level of the *IL-10* gene could influence *C*_0_/*D*. In this study, *IL-10* gene variants (*IL-10* rs1800871 C>T, *IL-10* rs1800872 C>A) presented a marginal association (*p*<0.05) with *C*_0_/*D* of renal recipients during the period of the predefined tacrolimus therapeutic range. However, the difference was not significant after correction by Bonferroni method. Since the Bonferroni method is very conservative, the effect of *IL-10* rs1800871 C>T and *IL-10* rs1800872 C>A on tacrolimus needs further investigation. In addition, six susceptible *COMT* variants and two susceptible *POR* variants were analyzed; however, none of these variants had a significant association with *C*_0_/*D*. Moreover, the variants of *CYP3A5 rs28365085 C*, CYP3A4*22 and *CYP3A4 rs33972239 delT* were not found in this study, although there are reports that they can affect tacrolimus pharmacokinetics [26]–[28]. This phenomenon revealed that the genetic background of tacrolimus metabolism varies among ethnic groups.

We examined the relationships between the five SNPs associated with the *C*_0_/*D* and the length of time required to reach the target *C*_0_ range. Of the five SNPs, *IL-10* rs1800871 C>T influenced the proportion of patients who achieved the target *C*_0_ range at weeks 3. MacPhee et al. reported that CYP3A5 nonexpressers achieved the target tacrolimus concentration easily, whereas there was a significant delay for CYP3A5 expressers [12]. In this study, there was no significant difference between the CYP3A5 expressers and CYP3A5 nonexpressers in the proportion of patients who achieved the target *C*_0_ range (Table 5). However, it appeared the genotypic groups with the higher *C*_0_/*D*, such as the *IL-10 rs1800871-TT* groups, were able to achieve the target *C*_0_ more easily. According to our data, the proportion of patients in the *IL-10 rs1800871-TT* group who achieved the target *C*_0_ range was higher (*p* = 0.004) compared to the *IL-10 rs1800871-CT* and *IL-10 rs1800871-CC* groups at week 3. A large proportion of patients achieved the target *C*_0_ range during week 3 after transplantation. So, it appears *IL-10* rs1800871 C>T was very important for the ease with which patients were able to achieve the target *C*_0_ range.

Owing to the strict inclusion and exclusion criteria, 97 patients with disease states that might affect tacrolimus pharmacokinetics were excluded. The exclusion of patients with some disease states is necessary because those diseases might affect tacrolimus metabolism and, thus, the results of the study. In addition, we selected days 1–3, 6–8 and 12–14 and the period of the predefined tacrolimus therapeutic range for analysis of the association between genetic polymorphisms and *C*_0_/*D*. Several time points were selected for the analysis, which was necessary because analysis of one genetic polymorphism at a single time point could produce an unreliable result.

There are several limitations to our study. The number of patients in several genotypic groups was small when the patients were divided into different groups according to genotype, which could influence the study results because of insufficient statistical power. Moreover, we can't confirm that CYP3A4*1G allele and *CYP3A4* rs4646437 T>C have independent effect on tacrolimus *C*_0_/*D*. The mechanism by which IL-10 affects the length of time required to reach the target *C*_0_ range is also unclear and further investigations are needed.

In clinical practice, the immunosuppressive effect of tacrolimus is not equivalent to tacrolimus *C*_0_. However, tacrolimus *C*_0_ is an important parameter to evaluate the immune status of transplant recipients. The latest insight into the genetic mechanism underlying tacrolimus metabolism has proved useful for tacrolimus individualization of organ transplantation patients. Some recent studies have individualized the dosage of tacrolimus on the basis of the *CYP3A5* genotype and obtained effective results [29], [30]. In this study, we found a significant association between tacrolimus *C*_0_/*D* and genotypes CYP3A5*3, CYP3A4*1G and *CYP3A4* rs4646437 T>C in Chinese renal transplant recipients. We observed increased proportions of patients with *IL-10 rs1800871-TT* genotypes who achieved the target *C*_0_ range. Therefore, genotyping of these genetic polymorphisms could potentially benefit Chinese renal transplant recipients by reducing the risk and the length of time needed to reach the target *C*_0_ range, and the results could be useful for the tacrolimus individualization of other organ transplant recipients.

## Supporting Information

Table S1 **Sample size and statistical power evaluation based on the different genetic variants.**
(DOC)
