# Supplementary material for: Impact of the CYP3A5, CYP3A4, COMT, IL-10 and POR Genetic Polymorphisms on Tacrolimus Metabolism in Chinese Renal Transplant Recipients
Source: PLoS One. 2014 Jan 21;9(1):e86206. doi: 10.1371/journal.pone.0086206 (PMC3897654; doi:10.1371/journal.pone.0086206)
Supplement: Table S1 — Sample size and statistical power evaluation based on the different genetic variants. (DOC) [file pone.0086206.s001.doc]

**Supporting information**

Table S1 Sample size and statistical power evaluation based on the different genetic variants.

|  |  | days1 to 3 | | days6 to 8 | | days 12 to 14 | |
| --- | --- | --- | --- | --- | --- | --- | --- |
|  |  | SDmax | SDmin | SDmax | SDmin | SDmax | SDmin |
| *CYP3A5*3* allele | Type-1 error | 0.05 | 0.05 | 0.05 | 0.05 | 0.05 | 0.05 |
|  | Numbers of group | 3 | 3 | 3 | 3 | 3 | 3 |
|  | Variance of means | 357.85 | 357.85 | 594.77 | 594.77 | 510.80 | 510.80 |
|  | Standard deviation | 53.20 | 35.10 | 62.40 | 27.10 | 59.30 | 32.70 |
|  | Effect size | 0.126 | 0.291 | 0.153 | 0.810 | 0.145 | 0.478 |
|  | Power(%) | 80 | 80 | 80 | 80 | 80 | 80 |
|  | Sample size (N) | 80 | 37 | 67 | 16 | 70 | 24 |
| *CYP3A4*1G* allele | Type-1 error | 0.05 | 0.05 | 0.05 | 0.05 | 0.05 | 0.05 |
|  | Numbers of group | 3 | 3 | 3 | 3 | 3 | 3 |
|  | Variance of means | 181.44 | 181.44 | 288.72 | 288.72 | 100.50 | 100.50 |
|  | Standard deviation | 53.20 | 32.10 | 62.20 | 37.30 | 59.70 | 35.8 |
|  | Effect size | 0.064 | 0.176 | 0.075 | 0.208 | 0.028 | 0.075 |
|  | Power(%) | 80 | 80 | 80 | 80 | 80 | 80 |
|  | Sample size (N) | 154 | 58 | 133 | 50 | 345 | 132 |
| *CYP3A4* rs4646437 T>C | Type-1 error | 0.05 | 0.05 | 0.05 | 0.05 | 0.05 | 0.05 |
|  | Numbers of group | 3 | 3 | 3 | 3 | 3 | 3 |
|  | Variance of means | 142.20 | 142.20 | 105.60 | 105.60 | 96.30 | 96.30 |
|  | Standard deviation | 52.30 | 29.20 | 60.90 | 25.60 | 57.80 | 12.80 |
|  | Effect size | 0.052 | 0.167 | 0.029 | 0.161 | 0.029 | 0.588 |
|  | Power(%) | 80 | 80 | 80 | 80 | 80 | 80 |
|  | Sample size (N) | 189 | 61 | 342 | 63 | 338 | 20 |

Abbreviations: SDmax, maximum standard deviation; SDmin, minimum standard deviation.

Note: The sample size and statistical power were evaluated according to the maximum standard deviation of C_0_/D and the minimum standard deviation of C_0_/D at the different times.
